# Effectiveness of percutaneous laser disc decompression versus conventional open discectomy in the treatment of lumbar disc herniation; design of a prospective randomized controlled trial

**DOI:** 10.1186/1471-2474-10-49

**Published:** 2009-05-13

**Authors:** Patrick A Brouwer, Wilco C Peul, Ronald Brand, Mark P Arts, Bart W Koes, Annette A van den Berg, Mark A van Buchem

**Affiliations:** 1 Department of Radiology, Leiden University Medical Center. Leiden, The Netherlands; 2 Department of Neurosurgery, Leiden University Medical Center, Leiden, The Netherlands; 3 Department of Neurosurgery, Medical Center Haaglanden, The Hague, The Netherlands; 4 Department of Medical Statistics, Leiden University Medical Center, Leiden, The Netherlands; 5 Department of general Practice, Erasmus University Medical Center, Rotterdam, The Netherlands

## Abstract

**Background:**

The usual surgical treatment of refractory sciatica caused by lumbar disc herniation, is open discectomy. Minimally invasive procedures, including percutaneous therapies under local anesthesia, are increasingly gaining attention. One of these treatments is Percutaneous Laser Disc Decompression (PLDD). This treatment can be carried out in an outpatient setting and swift recovery and return to daily routine are suggested. Thus far, no randomized trial into cost-effectiveness of PLDD versus standard surgical procedure has been performed. We present the design of a randomized controlled trial, studying the cost-effectiveness of PLDD versus conventional open discectomy in patients with sciatica from lumbar disc herniation.

**Methods/design:**

The study is a randomized prospective multi-center trial, in which two treatment strategies are compared in a parallel group design. Patients (age 18–70 years) visiting the neurosurgery department of the participating hospitals, are considered for inclusion in the trial when sciatica due to lumbar disc herniation has lasted more than 8 weeks. Patients with disc herniation smaller than 1/3 of the spinal canal diameter, without concomitant lateral recess stenosis or sequestration, are eligible for participation, and are randomized into one of two treatment arms; either Percutaneous Laser Disc Decompression or conventional discectomy. The functional outcome of the patient, as assessed by the Roland Disability Questionnaire for Sciatica at 8 weeks and 1 year after treatment, is the primary outcome measure. The secondary outcome parameters are recovery as perceived by the patient, leg and back pain, incidence of re-intervention, complications, quality of life, medical consumption, absence of work and secondary costs.

**Discussion:**

Open discectomy is still considered to be the golden standard in the surgical treatment of lumbar disc herniation. Whether Percutaneous Laser Disc Decompression has at least as much efficacy as the standard surgical procedure, and is more cost-effective, will be determined by this trial.

**Trial registration:**

Current Controlled Trials ISRCTN25884790.

## Background

In the majority of patients, experiencing their first episode of sciatica due to lumbar disc herniation, the symptoms recede to a non-disabling level within a period of six weeks [1]. The historical mainstay in the treatment of sciatica in patients in which the complaints are refractory to conservative treatment is discectomy [2]. This treatment is aimed at the removal of the herniated disc fragment that is the cause of nerve root compression. Another way of decompressing the nerve root is by inducing a negative pressure in the intervertebral disc by removal of tissue. Several percutaneous techniques are based on this principle [3]. Percutaneous Laser Disc Decompression (PLDD), being one of these techniques, is a modality in which laser energy is delivered to the nucleus pulposus by means of a fiber [4]. This fiber is inserted through a thin needle via a posterolateral percutaneous approach under local anesthesia. The absorption of the applied laser energy leads to vaporization of the water content of the nucleus pulposus in combination with a change in protein structure thereof. The subsequent volume reduction causes a disproportionate decrease in intradiscal pressure and relieves the nerve root. The first clinical percutaneous laser disc decompression was performed in Europe by Choy and Ascher in 1986 [5]. The U.S. Food and Drug Administration approved PLDD for use in the USA in 1991.

PLDD is an attractive treatment because of the minimally invasive nature and therefore the assumed decrease in risk of structural damage to the muscles, bone, ligaments and nerves. Furthermore, the patients are expected to have less back pain, shorter hospitalization and a shorter reconvalescence period than with conventional surgery. The actual recovery of the sciatica however, might take more time than after conventional surgery, although immediate resolution of the symptoms does occur.

Although several cohort studies have been published, and FDA approval was given, to date no randomized trial has been performed comparing PLDD with conventional surgical procedures. The cohort studies showed the safety and potential benefits of PLDD. No evidence regarding the efficacy and cost-effectiveness of PLDD, compared to conventional surgery, is available to date [6].

Currently, there is broad consensus that conventional surgery is the gold standard for surgical intervention for sciatica; therefore PLDD has to be compared to conventional surgery in order to assess the cost-effectiveness. The result will be a trade-off between the expected more swift recovery of the patients in the 'gold standard' group versus the minimally invasive nature, lower costs and patient comfort of the PLDD treatment, which might take a slightly longer period to full recovery. Several subgroups will be identified in a post-hoc analysis, using pre-defined potential risk factors, which might especially benefit from the PLDD treatment. It is hypothesized that the potential result, immediately after treatment, is non-inferior at longer follow-up. In this study the patients will therefore be assessed for primary endpoints at 8 weeks and 1-year follow up and additional endpoints up to 2 years.

## Methods/Design

We designed a randomized prospective open trial aimed at showing non-inferiority of PLDD to the surgical treatment of lumbar disc herniation. Both treatments are compared in a parallel group design with at least a 2-year follow-up. The Roland Disability Questionnaire for Sciatica will be used as the primary outcome measure to determine the power of the study. A multi-center approach is used to assure a sufficient recruitment of participating patients. In this case 2 academic hospitals and 6 teaching hospitals will recruit patients. We received full approval of the ethics committees in all participating hospitals.

### Patients

**Figure 1:**
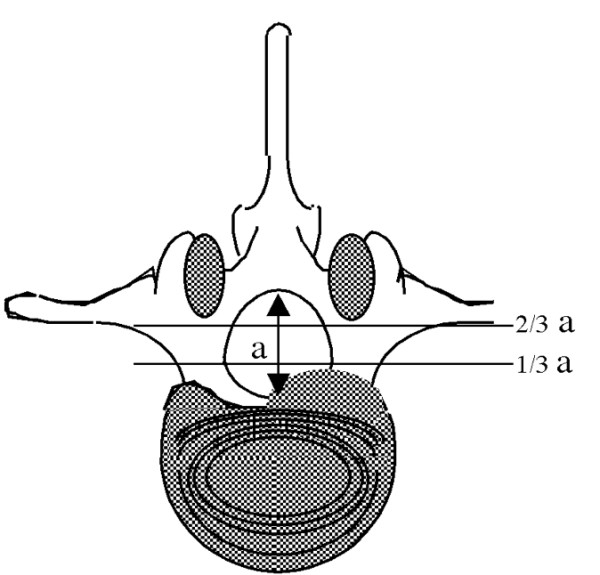
**Measuring the disk herniation**. The size of the herniated disc is measured in relation to the spinal canal diameter at disc level. The size should not exceed one third of the total spinal canal diameter.

All patients between 18 and 70 years of age with sciatica since 6–8 weeks, presenting at the neurosurgical department, are eligible for inclusion in the trial. A disc herniation at the appropriate level will have to be shown by MRI. The herniated fragment has to be smaller than 1/3 of the spinal canal (figure 1). The remainder of the patients (i.e. those with a herniated fragment > 1/3 of the spinal canal) is allocated to a trial running simultaneously studying Micro Endoscopic Discectomy (MED) versus conventional surgery [7]. Patients' history and standard neurological examination will be documented by the neurosurgeon, who subsequently decides on the eligibility of the patient for the trial, based on Table 1, and explains the trial to the patient. If the patient is willing to participate, an appointment is made with one of the research nurses. This visit is planned at least 2 days later to give the patient ample time to decide on entering the study. After informed consent the research nurse records the baseline variables, questionnaires and outcome measures.

**Table 1:** Selection criteria for patient eligibility

Inclusion:
*Age 18–70 years
*Persistent Radicular pain lasting more than 6–8 weeks
*Operation indication
*Disc herniation confirmed at MRI
*Unilateral disc herniation smaller or equal to 1/3 of the spinal canal
*Informed consent
Exclusion:
*Previous surgery at the same disc level
*Cauda equina Syndrome
*Spondylolytic or degenerative spondylolisthesis
*Central spinal canal stenosis
*Pregnancy
*Severe somatic or psychiatric illness
*Planned (e)migration to another country in the year after the inclusion
*Inadequate verbal or writing skills in Dutch language

### Randomization procedure

Patients will be randomly allocated to PLDD or conventional surgery. This randomization will take place during the outpatient visit with the research nurse following the informed consent procedure, but after completion of the baseline assessments. A randomization list is prepared for every participating hospital/nurse combination. Variable sized blocks of random numbers are formed to ensure equal distribution of the randomization treatments over hospitals and research nurses. The data manager at the department of Biostatistics, who is not involved in the selection and allocation of patients, will prepare coded, sealed envelopes containing the treatment allocation. The patient will open the envelope in the presence of the research nurse.

### Intervention

Patients will be allocated to either discectomy (A) or PLDD (B), which will take place within 4 weeks after inclusion.

#### (A) Open discectomy

Depending on the surgeon and patient's preference, surgery will be performed under general or spinal anesthesia. The patients will be positioned prone and the affected disc level is verified with fluoroscopy. A small midline incision (2–3 cm) will be made and the paravertebral muscles will be dissected unilaterally. Laminotomy will be performed when deemed necessary. In order to decompress the nerve root, the herniated disc will be removed as much as possible through a unilateral transflaval approach. The wound will be closed in layers with a suction drain when necessary. Patients will be operated with loupe magnification or microscope depending on the surgeon's preference. The participating surgeons have large experience in the technique. A standardized case record form (CRF) will register the surgeon's findings and will be send to the data center. Patients will be admitted to hospital for 2–7 days depending on the usual care.

#### (B) Percutaneous Laser Disc Decompression

The patient will be instructed to take a prone position on the table of the CT-scan. After placing sterile drapes the level of treatment is identified by a scan. The needle entry point is anesthetized by local lidocaine injection no deeper than the facet joint. Subsequently the 18G needle is placed centrally in the nucleus pulposus, and parallel to the endplates by means of a posterolateral approach. Through the needle, a glass fiber is placed in the disc, enabling the application of laser energy (980 nm, 7 W, 0,6 s pulses, interval 1 second). After a total energy of 1500 J is delivered (2000 J for level L4-5), the procedure is finished. A control CT scan is performed to assess gas formation in the disc space. After the treatment the patient is allowed to drink tea or coffee and is observed for 15 minutes before he/she can return home.

Both treatment strategies will be followed by active mobilization in the post-operative period. Patients in the surgery group will be discharged as soon as possible. Early resumption of daily activities and work will be stimulated in both study groups.

### Baseline data

At baseline we will register demographics, hobbies, sports, work status, smoking status, back pain history, family history of sciatica, co-morbidity, patients satisfaction at work, weight and length. The treatment preference of both patient and the including surgeon will be noted on a 5-point scale ranging from "strong preference for conventional open discectomy" to "strong preference for PLDD". We will also register the expected recovery at 8 weeks after randomization as expected by both surgeon and patient. The baseline questionnaires for the primary endpoints are also collected.

### Outcome assessment

The assessment of outcome will use several validated outcome parameters as described below. Patients will undergo neurological examination by the research nurse at 4, 8, 26, and 52 weeks (Table 2). Questionnaires will also be filled out at these follow-up moments. At 1, 2, 6, 12, 38, 78, and 104 weeks the questionnaires will be filled out by the patient at home, and subsequently send to the data collection center by regular mail. Outpatient control by the neurosurgeon takes place at 8 weeks and thereafter if deemed necessary. The follow-up moments of data collection and outcome measures are listed in Table 3.

**Table 2:** Neurological examination

*Straight leg raising test (Lasègue)
*Crossed straight leg raising test (Crossed Lasègue)
*Sensory loss
*Dermatome anesthesia
*Muscle weakness
*Knee tendon reflex difference
*Ankle tendon reflex difference
*Finger-ground distance in centimeter

Lasègue's sign is defined positive if the patient experiences a typically dermatomal area of pain reproduction and pelvic muscle resistance below a unilateral 60 degrees angle provocative straight leg raising. 'Crossed positive' if the same experience was noted with straight raising of the contralateral leg below 90 degrees.

**Table 3:** Data collection and outcome measures

	RN			RN		RN		RN		RN		
**Time in weeks**	***X***	0	2	***4***	6	***8***	12	***26***	38	***52***	78	104

Treatment preference patient	v	v								v		v

Neurological examination	v			v		v		v		v		

Likert	v	v	v	v	v	v	v	v	v	v	v	v

Prolo	v			v		v		v		v		

Severity of complaints (VAS)	v	v	v	v	v	v	v	v	v	v	v	v

Functional status (Roland DQS)	v	v	v	v	v	v	v	v	v	v		v

McGill	v									v		v

Health status (Rand/SF 36)	v			v		v		v		v	v	v

EuroQol	v	v	v	v	v	v	v	v	v	v	v	v

SFB Index	v			v		v		V		v		

Costs (diary)	v		v	v		v	v	V	v	v		

Surgery/PLDD		v										

Complications		v		v		v		v		v		

"RN" marks the scheduled meetings with the Research Nurse.

Our goal is to show clinical equivalence between the two treatment strategies at various time points. The 1-year follow-up will be the most important clinical landmark. The cost-effectiveness analysis will therefore be performed using the clinical and economical parameters at 1 year.

### Primary outcome measure

Roland Disability Questionnaire for Sciatica (RDQ) is an illness-specific 23-item functional assessment questionnaire that is frequently used for low back pain and sciatica [8-11]. The score, ranging from 0 to 23, reflect a sum of items experienced by the patient. The higher the score the more disabling the sciatica is perceived. The score will be analyzed as a continuous outcome variable. For descriptive purposes, "Recovery" (at the individual level) is defined by a difference of at least 11 points from baseline. A difference of 4 points for the averages between the two randomization arms is considered the maximum for "equivalence" of the two treatment modalities. The RDQ has established validity, reliability and responsiveness to change [12]. This outcome measure is used to determine the power of the study.

Other parameters that will be used from primary endpoint analyses are:

#### 1) Perceived recovery

This is a 7-point Likert scale measuring the perceived recovery, varying from 'complete recovery' to 'worse than ever'. This outcome scale has been used in previous studies and is regarded valid and responsive to change [13]. We will also score a job- and hobby specific Likert in which the patient will be asked to rank their 5 most important functional disabilities which will be used in their own evaluation overall and in separate items.

#### 2) Visual Analog Scale (VAS) of leg pain

The experienced pain intensity in the leg will be assessed at fixed intervals on a visual analog scale. The scale will measure 100 mm varying from 0 mm 'no pain' to 100 mm 'the worst pain imaginable'. The VAS has shown reliability, validity and responsiveness [14].

#### 3) Visual Analog Scale (VAS) of back pain

Many of the patients have, in addition to their radicular pain, back pain as well. The back pain will be assessed by means of a VAS

Additional (secondary) outcome measures

#### 1) McGill pain questionnaire

Perceived pain will be measured by using the Dutch version of the validated McGill Questionnaire [15].

#### 2) Short-form 36 (SF 36)

The RAND-SF 36 is a generic health status questionnaire that consists of 36 items on physical and social status of the patient subdivided in 8 domains. These are physical functioning, physical restrictions, emotional restrictions, social functioning, somatic pain, general mental health, and vitality and general health perception. The questions add up to a score of 0 (worst health) to 100 (ideal health). The SF 36 has been validated for surgical studies on low back pain pathology [16-18].

#### 3) Sciatica Frequency and Bothersome Index (SFBI)

This scale, ranging from 0 to 6, can asses frequency (0 = never, 6 = always) and bothersome (0 = not at all, 6 = extreme bothersome) of back and leg pain. The sum of the questions ranges from 0 to 24 [8,19].

#### 4) Prolo scale

The Prolo scale measures the evaluation of the surgeon and research nurse of the patient's functional and economic status. It is a current scale in outcome studies of lumbar spinal surgery [20,21].

#### 5) Costs

Direct medical costs of hospital admission and surgery as well as PLDD related costs will be based on an integral cost-analysis in the participating hospitals. From this analysis, the constant costs per treatment and the variable costs will be estimated. Other medical costs (physiotherapy, general practitioner, nursing care, medication and medical specialists) will be registered in a diary. Non-medical costs (time, travel expenses, domestic help) will also be included in the diary. The research nurse will go through the diary with the patient on every follow-up moment throughout the first 2 years. To estimate the indirect costs the patient will register absenteeism. The research nurse will register the work situation, work efficiency and gross income on the follow-up moments. Absenteeism will be valued to the friction-cost method.

#### 6) Incidence of re-operations

The incidence of re-operation will be used as an outcome measure.

#### 7) Complications

Complications will be registered systematically. This includes wound infection, deep venous thrombosis, urine tract infection, hematoma and progressive neurological deficit. The surgeon and research nurse will register the complications at the follow-up moments.

### Sample size

The study is designed as an equivalence study with asymmetric boundaries, leading to a non-inferiority design. A difference of 4 on the Roland Disability Questionnaire has been recognized as the minimum clinically important difference. The power calculation is performed under the alternative hypothesis of a difference of 1 point on the Roland scale. By using this value 4 as the upper limit of the equivalence interval, with α-level of 0.05, a power of 0.90 and a SD of 5, the required sample size was 98 (49 per treatment arm). To adjust for an 8% loss to follow up, we plan to include 110 patients. These calculations are based on a comparison of average at single points in time. By using a repeated measurements analysis of variance, the power of the study over the entire follow-up period will be substantially higher (or equivalently, the average difference between the treatments can be established more accurately).

### Statistical analysis

Baseline comparability between the two treatment groups will be evaluated with respect to disease characteristics, demographics and baseline values of self-report measures. Chi-square or Student t-test, as appropriate, will be used for these comparisons, although decisions to adjust for one or more of the base line characteristics in the actual analyses will not be based on statistical significance but on the relevance of the confounding effects in case of imbalance occurring in spite of the balanced randomization procedure. The analysis will be performed in accordance with the "intent-to-treat principle", analyzing all patients within their randomization groups, regardless of whether they completed the allocated treatment. Intent-to-treat analysis often leads to smaller observed treatment differences, thereby increasing the risk of type I error (claiming there is non-inferiority/equivalence, when there is not). Therefore, a per-protocol analysis is also carried out and differences in results will be described.

The analysis will consist of assessment of differences in outcome measurements between both groups and differences in time to recovery. It will take into account the stratification factors (center; research nurse) as covariates in accordance with ICH E9. Other base line covariates, measured before randomization, may be incorporated into the analysis models to increase power and to remove any residual confounding. An assessment of interaction between the treatment on the one hand and the stratification variables, or. main baseline covariates, will be considered as a mandatory part of the primary analysis and a proper interpretation of efficacy. The "difference" between the two treatment arms, the treatment effect (on the appropriate scale) will be reported both as an overall (adjusted) value and – in the case of significant effect modification – in terms of its dependence on the categories or values of the effect modifiers. A significant interaction between the stratifying factors or main baseline covariates will be considered as part of a proper modeling strategy and will not imply correction for repeated significance testing. After fitting the proper model, inference on the treatment effect will be based on the smallest fitting model that allows estimation of the treatment effect.

When analyzing the primary outcome measure, Roland, the primary analysis will be a repeated measurement analysis of variance, hence estimating the original measurements. As a secondary analysis of this primary outcome variable, we will define "Roland-recovery" as a change of at least 11 points between baseline and any point in time post randomization. This dichotomization of the Roland score will be used as the outcome variable in a Cox regression analysis modeling time till recovery. The multivariate analysis will be performed along the same principles for variable selection and model building as outlined above.

A subgroup analysis is preplanned and will be performed to assess differences in treatment effect in subgroups of patients. (Table 4)

**Table 4:** Prognostic variables in subgroup analysis

**Demographic variables:**
*Age < 40 years versus > 40 years
*Higher education versus lower education
*Non-physical versus physically demanding jobs
**Anamnestic variables and neurological variables**
*Quetelet index < 25 versus > 25
*Influence of sitting on complaints versus no influence
*Straight leg raising < 30° versus low-back pain
**MRI variables**
*Median versus mediolateral and lateral herniation
*High disc space versus low disc space
*Higher signal versus lower signal of disc on T2 weighted images
*Bud-herniation versus broad based herniation

Data will be stored via the internet-based secure data management system (ProMISe) of the department of Medical Statistics and Bioinformatics. The analysis will be carried out using SPSS.

## Discussion

With this publication we introduce the design of the first randomized controlled trial on the cost-effectiveness of PLDD versus conventional surgical discotomy for sciatica in lumbar disc herniation. This randomized controlled trial is a prospective trial comparing the effects of the techniques as well as the economical costs. The aim is to investigate whether both treatment modalities are equivalent in their clinical effect and to study their cost-effectiveness.

The inclusion is complete as off the end of 2007 with follow-up measurements going on until at least the end of 2009.

## Competing interests

The authors declare that they have no competing interests.

## Authors' contributions

PAB is the coordinator and principal investigator of the trial and is responsible for data management of the trial. He designed the study protocol together with WP, BK and AvdB. MA is co-principal investigator of the parallel MED-trial [7], and co-wrote this manuscript. RB has contributed to the case record forms, is the responsible biostatistician and is responsible for the ProMISe software providing the data management infrastructure for the trial. MvB is the neuroradiological supervisor. All authors participated in the trial design and coordination. All authors read and approved the final manuscript.

## Pre-publication history

The pre-publication history for this paper can be accessed here:


